# Wastewater-based surveillance identifies start to the pediatric respiratory syncytial virus season in two cities in Ontario, Canada

**DOI:** 10.3389/fpubh.2023.1261165

**Published:** 2023-09-26

**Authors:** Elisabeth Mercier, Lakshmi Pisharody, Fiona Guy, Shen Wan, Nada Hegazy, Patrick M. D’Aoust, Md Pervez Kabir, Tram Bich Nguyen, Walaa Eid, Bart Harvey, Erin Rodenburg, Candy Rutherford, Alex E. Mackenzie, Jacqueline Willmore, Charles Hui, Bosco Paes, Robert Delatolla, Nisha Thampi

**Affiliations:** ^1^Department of Civil Engineering, University of Ottawa, Ottawa, ON, Canada; ^2^Hamilton Health Sciences, McMaster Children’s Hospital, Hamilton, ON, Canada; ^3^Research Institute, Children’s Hospital of Eastern Ontario, Ottawa, ON, Canada; ^4^Hamilton Public Health Services, Hamilton, ON, Canada; ^5^Hamilton Regional Virology Laboratory, Hamilton, ON, Canada; ^6^Department of Pediatrics, Children’s Hospital of Eastern Ontario, Ottawa, ON, Canada; ^7^Department of Pediatrics, University of Ottawa, Ottawa, ON, Canada; ^8^Ottawa Public Health, Ottawa, ON, Canada; ^9^Department of Pediatrics (Neonatal Division), McMaster University, Hamilton, ON, Canada

**Keywords:** respiratory syncytial virus, wastewater-based surveillance, pediatric hospitalization, community incidence, season start date, active immunization, palivizumab, hospital-level preparedness

## Abstract

**Introduction:**

Detection of community respiratory syncytial virus (RSV) infections informs the timing of immunoprophylaxis programs and hospital preparedness for surging pediatric volumes. In many jurisdictions, this relies upon RSV clinical test positivity and hospitalization (RSVH) trends, which are lagging indicators. Wastewater-based surveillance (WBS) may be a novel strategy to accurately identify the start of the RSV season and guide immunoprophylaxis administration and hospital preparedness.

**Methods:**

We compared citywide wastewater samples and pediatric RSVH in Ottawa and Hamilton between August 1, 2022, and March 5, 2023. 24-h composite wastewater samples were collected daily and 5 days a week at the wastewater treatment facilities in Ottawa and Hamilton, Ontario, Canada, respectively. RSV WBS samples were analyzed in real-time for RSV by RT-qPCR.

**Results:**

RSV WBS measurements in both Ottawa and Hamilton showed a lead time of 12 days when comparing the WBS data set to pediatric RSVH data set (Spearman’s ρ = 0.90). WBS identify early RSV community transmission and declared the start of the RSV season 36 and 12 days in advance of the provincial RSV season start (October 31) for the city of Ottawa and Hamilton, respectively. The differing RSV start dates in the two cities is likely associated with geographical and regional variation in the incidence of RSV between the cities.

**Discussion:**

Quantifying RSV in municipal wastewater forecasted a 12-day lead time of the pediatric RSVH surge and an earlier season start date compared to the provincial start date. These findings suggest an important role for RSV WBS to inform regional health system preparedness, reduce RSV burden, and understand variations in community-related illness as novel RSV vaccines and monoclonal antibodies become available.

## 1. Introduction

Human respiratory syncytial virus (RSV) is a major cause of lower respiratory tract infections, with a disproportionate impact on both children less than six months of age and those with complex medical conditions (1–3). Globally, RSV is associated with approximately three million hospitalizations and over 100,000 deaths annually in children younger than five years, with 99% mortality occurring in low- and middle-income countries (4). Even in well-resource settings, RSV is the leading cause of hospitalizations among infants aged zero to six months, who comprise nearly 40% of the burden (5). In Canada, the RSV season typically starts in November, peaks in December through January, and ends in March or early April. During this period, approximately 1% of all infants in their first year of life are hospitalized due to RSV. However, in more remote communities, this rate significantly increases, affecting 20 to 50% of all live births (6).

As with almost all jurisdictions worldwide (7), RSV testing in Canada is restricted to symptomatic children who present to the emergency room and are likely to require hospitalization (8). Trends of pediatric and adult test positivity are the main source of near-real-time public health surveillance, yet this metric is a known lagging indicator of infection prevalence in communities which may impact optimal hospital-level preparedness and the timely initiation of immunologic protection for at-risk children in the community. As such, current clinical surveillance of RSV significantly underrepresents the prevalence of RSV and extent of pediatric infections in the community (6).

The severity of RSV infection in premature infants and children with existing comorbidities can be significantly reduced by timely immunoprophylaxis with palivizumab, a human monoclonal antibody (IgG1κ), administered ideally just prior to the start of the RSV season and every 30 days throughout the RSV season (9). In Ontario, the RSV season start and end dates are declared according to clinical criteria, after which specific clinical sites are authorized to provide government-funded RSV immunoprophylaxis for children under age 2 years at highest risk of severe disease. Specifically, the RSV season start date is typically identified by the Ontario Ministry of Health based on an increase in provincial pediatric RSV-positive specimens reported by the Laboratory Respiratory Pathogen Surveillance Report from Public Health Ontario (10). Similarly, the end date is determined by the resolution of provincial pediatric RSV-positive specimens identified. There is currently no pre-defined threshold of RSV percent positivity to identify the start and end dates of the RSV season; however, pathogen trends in geographically distinct areas of Ontario are reviewed by an expert advisory panel to inform decisions around initiation and duration of RSV immunoprophylaxis in specific pediatric populations. Despite this strategy, RSV infections continue to result in substantial healthcare costs (11). Current clinical surveillance could be enhanced with additional tools that offer early warning signals of RSV transmission in communities and thus offer the means to more accurately define the RSV season’s start and end dates. Wastewater-based surveillance (WBS) is one such promising tool.

The SARS CoV-2 pandemic has underscored the value of accurate, real time, and sensitive surveillance for the early detection and monitoring of a circulating pathogen in the community (12). WBS of viral-specific biomarkers shed by infected individuals was implemented two decades ago for the early detection and monitoring of enteric pathogens, such as poliovirus (13). It has gained recognition over the last 3 years as a cost-effective means of monitoring disease incidence at the population level while minimizing invasive procedures (2, 14). Additionally, WBS was shown to facilitate identification of communicable disease hotspots by public health, such as COVID-19 and its variant, influenza and poliovirus which further led to demand for inclusion of other pathogens in the WBS frameworks (15, 16). Moreover, previous efforts with respiratory pathogen surveillance in wastewater has positioned the WBS system to track the community level incidence of seasonal viral diseases like RSV more efficiently, equitably, and comprehensively than current clinical testing practice (2, 17, 18).

The aims of this study are to assess the applicability of WBS for the early detection of RSV community transmission to provide a valuable delineation of the onset of the pediatric RSV season, while enabling more timely initiation of individual immunoprophylaxis and broader public health and hospital-based measures. As such, we conducted RSV wastewater surveillance as a means of early identification and more accurately defining the RSV season start and end dates to help guide palivizumab administration and hospital preparedness in two cities in Ontario, Canada.

## 2. Materials and methods

### 2.1. Site descriptions and wastewater sample collection and processing

In Ottawa, wastewater samples were harvested from the Robert O. Pickard Environmental Centre, the sole water resource recovery facility (WRRF) in city, which serves approximately 910,000 individuals, 91% (910,000/1,000,000) of Ottawa’s population (Supplementary Table S1). In Hamilton, wastewater samples were harvested from the Woodward Avenue Treatment Plant, serving approximately 512,000 individuals, again 91% (512,000/561,780) of the city’s population (Supplementary Table S1). The cities of Ottawa and Hamilton were chosen because of similarities in population coverage served by their respective WRRF, the population mean age (Supplementary Table S1) and having a sole pediatric acute care facility serving their respective regions. In addition, the cities were geographically distant enough (Supplementary Figure S1) to not have overlapping patient populations informing the start of their respective RSV season.

Primary clarified sludge was manually collected every 6 hours by operators and combined into a 500 mL, 24-h composite sample from Ottawa’s and Hamilton’s WRRFs between August 1, 2022, and March 5, 2023, to cover the entirety of the RSV season in Ontario. The samples were collected daily for Ottawa and five times per week for Hamilton, due to resource limitations. Both sampling frequency is above the recommended baseline for actionability of three samples per week (19). Following collection, the primary sludge samples were immediately refrigerated at 4°C for transport to the laboratory for analysis. All samples were processed within 24-h of reception. Forty milliliters of well-homogenized primary sludge WRRF samples was centrifuged, and 250 mg of the pelleted material was processed using the RNeasy PowerMicrobiome (Qiagen) methodology as previously described (17, 20).

### 2.2. Wastewater sample analysis

RSV and pepper mild mottle virus (PMMoV), an endogenous human fecal biomarker, were measured by singleplex, reverse transcription quantitative real-time polymerase chain reaction (RT-qPCR; Bio-Rad, Hercules, CA) using previously developed assays (21). PMMoV measurements were used to monitor for RT-PCR inhibition as well as to correct for significant snowmelt periods or rainfall events. RSV wastewater levels are presented as a mass fraction of the RSV signal, where copies of RSV are normalized per gram of wastewater solids. Mass fraction units are used in this study as preliminary tests, which align with the literature (2), indicated that RSV RNA preferentially partitions to the solid portion of wastewater.

All primers and probes, RT-qPCR cycling conditions, and reagent concentrations are described in Supplementary Tables S2, S3. Each sample was run in triplicate with non-template controls and a five-point standard curve, prepared with a reverse transcriptase digital droplet PCR (RT-ddPCR)-quantified RSV G-block (Supplementary Table S2) and endogenous PMMoV from wastewater extract. The assay’s potential for cross-reactivity in wastewater was assessed by spiking the wastewater with respiratory analytes (using Exact Diagnostics Respiratory Positive Run Control) at different concentrations. The specificity of the amplicon was verified through Sanger sequencing with all tests exhibiting a homology greater than 95% to RSV reference sequence (hRSV/A/England/397/2017|EPI_ISL_412866|2017-01-01). The assay’s limit of detection (ALOD) and limit of quantification (ALOQ) for RSV’s targeted N gene region (Supplementary Table S3) was approximately 3.0 and 6.0 copies/reaction, respectively. RT-qPCR efficiency ranged from 90–110% and the coefficient of determination (R2) values were greater than 0.98 (*n* = 233) (22).

### 2.3. Clinical data

Daily pediatric laboratory-confirmed RSV hospitalization (RSVH) admissions were collated weekly from the Children’s Hospital of Eastern Ontario for the city of Ottawa and McMaster Children’s Hospital for the city of Hamilton. Both institutions are the sole designated pediatric acute care facilities for infants and children admitted from the region. Patients with nosocomial RSV infection, defined as a positive RSV test identified more than 72 h after admission, were excluded from the study.

Weekly regional RSV case data were also used in this study. Regional case data was collected from Ottawa and its surrounding area (Eastern Ontario Regional Laboratory Association for Ottawa), in addition to Hamilton and its surrounding area (Hamilton Regional Virology Laboratory for Hamilton). Regional RSV case data includes adult and pediatric inpatient and outpatient cases in both cities and their surrounding areas. In both cities, the clinical criteria for RSV (along with Influenza A and B) qPCR-based testing in adults and children are; admission to hospital with a respiratory-related infection, and out patients at increased risk of influenza-associated complications who are eligible for chemoprophylaxis with oseltamivir (23). There were no changes in the testing criteria or clinical practices for patients with suspected or confirmed RSV within the Ottawa or Hamilton regions during the COVID-19 pandemic or during the autumn 2022/2023 viral respiratory season period.

### 2.4. Definition of clinical start and end of RSV season

The RSV season start date for the province of Ontario, which includes both the cities of Ottawa and Hamilton and their surrounding regions, is typically identified by the Ontario Ministry of Health based on an increase in provincial pediatric RSVH reported by the Laboratory Respiratory Pathogen Surveillance Report from Public Health Ontario (10). Similarly, the end date is determined by the resolution of provincial pediatric RSVH. There is currently no pre-defined threshold of RSVH or percent positivity data to identify the start and end dates of the RSV season; however, pathogen trends in geographically distinct areas of Ontario are reviewed by an expert advisory panel to inform decisions around initiation and duration of RSV immunoprophylaxis in specific pediatric populations.

### 2.5. Statistical analyses

A stepwise comparison with incremental 24-h offsets of the wastewater RSV measurements versus RSVH data was used to identify the lead time of the WBS data that resulted in the strongest WBS-clinical correlation (24). Visual alignment of peaks of the WBS data and RSVH data sets in conjunction with highest Spearman’s rank correlation coefficient (*ρ*) was utilized to confirm the lead times of the WBS data sets as previously described (24). The Spearman’s rank correlation coefficient calculations were done with a significance level (α) of 0.05. The analysis was conducted on data sets that ranged from August 1^st^ to November 30, 2022, with this range of dates enabling the study to focus on the onset and resolution of the RSV season. Time-step Spearman’s Rank correlation analyses were performed, using GraphPad Prism (version 9.3.1), as non-normality of the data was established using a quantile-quantile plot.

### 2.6. Ethical considerations

The study was submitted for review to the University of Ottawa, the Children’s Hospital of Eastern Ontario Ethics Boards as well as the Hamilton Integrated Research Ethics Board. It was determined that the use of wastewater-acquired viral signals at citywide levels and the secondary use of anonymized, aggregated clinical data did not require the board review and approval. The study was also approved by the Ottawa and Hamilton Public Health research ethics committees.

## 3. Results

### 3.1. Lead time of RSV WBS compared to clinical data

**Figure 1:**
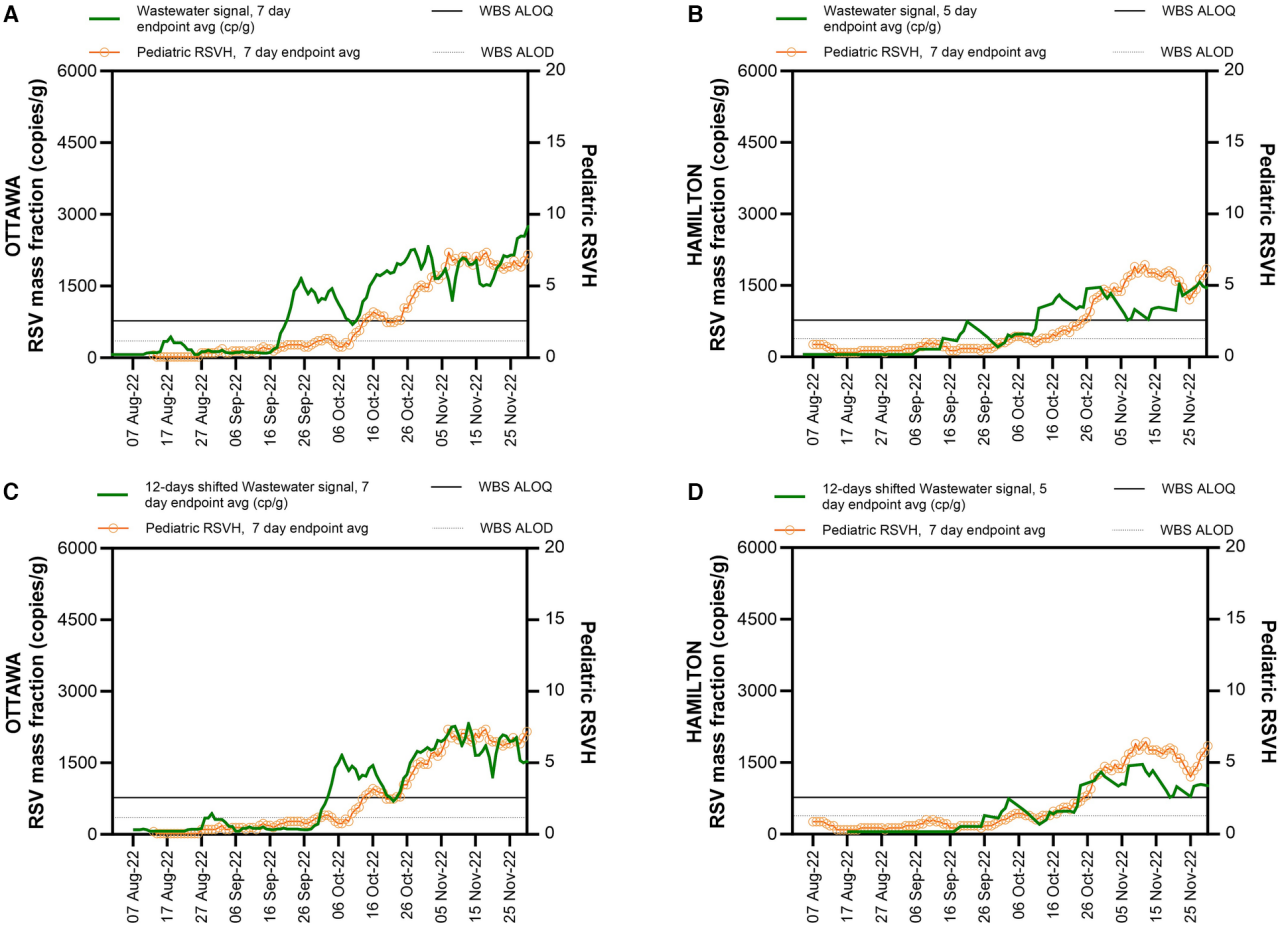
WBS RSV measurements and pediatric RSVH during RSV season onset. WBS RSV measurements (gene copies per grams of wastewater solids) and pediatric RSVH from August 1^st^ to November 30, 2022, **(A)** in Ottawa. **(B)** in Hamilton. **(C)** 12-day shifted WBS RSV measurements for Ottawa. **(D)** 12-day shifted WBS RSV measurements for Hamilton. The solid horizontal line indicates the WBS assay limit of quantification (ALOQ) and the lower horizontal dotted line indicates the WBS assay limit of detection (ALOD).

**Figure 2:**
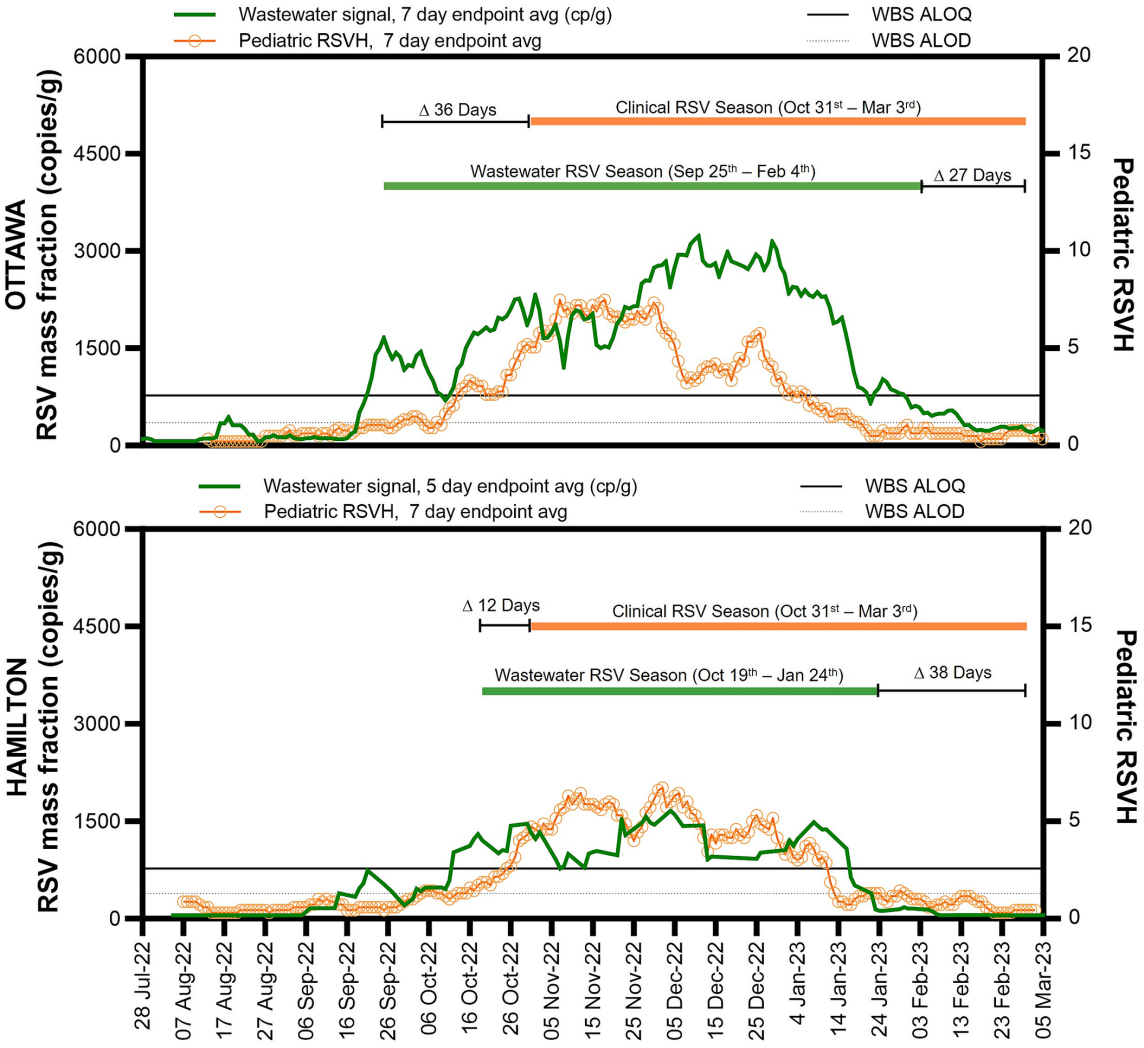
WBS RSV and pediatric RSVH surveillance data. WBS RSV measurements (gene copies per gram of wastewater solids) and pediatric RSVH from August 1st to March 5, 2023, for Ottawa (upper graph) and Hamilton (lower graph). The solid horizontal line indicates the ALOQ and below that the horizontal dotted line indicates the ALOD.

During the study period, RSV wastewater signal was first detected in the city of Ottawa on August 11, followed by the city of Hamilton on September 7, 2022 (Figures 1A,B). An RSV wastewater signal was detected in 84% (177/210) of Ottawa city samples and in 54% (63/117) of those from the city of Hamilton. During the same period, a mean of 436 (± 201) clinical PCR-based RSV tests were performed weekly in Ottawa, with a peak of 908 tests during the first week of December (Figure 2). In Hamilton, the average number of weekly tests performed was 744 (± 151), reaching a maximum of 990 tests during the first week of January (Figure 2). The first pediatric RSVH was on August 7 and August 4, 2022, in the cities of Ottawa and Hamilton, respectively, with increasing numbers observed through October and into November (Figures 1A,B). Offsetting the WBS data forward in one-day increments up to 25 days, showed that a 12-day forward shift of WBS data generated the highest Spearman’s correlation (ρ =0.90) (Supplementary Table S4). The 12-day forward shift also yielded the best peak alignment between the WBS and RSVH data sets in the two cities as shown in Figures 1C,D.

The proposed start of the RSV season based on WBS data was defined as one week of consecutive endpoint average RSV wastewater measurements (copies/g wastewater solids) above the ALOQ (Figure 2). For the city of Ottawa, this corresponded to seven consecutive days of the seven-day endpoint average above the ALOQ. In Hamilton, the collection of wastewater samples was performed at a frequency of five days a week; thus, the criterion was adjusted to be five consecutive days of the five-day endpoint average RSV signal (copies/g wastewater solids) above the ALOQ. The criteria used in this study was adapted from Zhao et al. for WBS of SARS-CoV-2, who proposed using three consecutive increasing data points when sampling twice a week to identify an increasing trend in WBS data (25).

### 3.2. Lead time of WBS identified start of RSV season compared to clinical data

Following surveillance reports of increasing pediatric RSVH and RSV test positivity across the province, the Ontario Ministry of Health declared that the 2022 RSV season in the province started on October 31, 2022, which enabled clinical sites to begin government-funded RSV immunoprophylaxis for high-risk infants; the end date was declared as March 3, 2023 (26). The RSV WBS season start, defined as one week of consecutive endpoint average RSV measurements above the ALOQ, was notably different between the city of Ottawa and Hamilton and preceded the clinically-determined provincial start date by 36 days (September 25, 2022) in the city of Ottawa and 12 days (October 19, 2022) in the city of Hamilton (Figure 2). By December 1, 2022, pediatric RSVH in Ottawa began to decline and diverge from the 12-day shifted RSV WBS measurements, with the RSV WBS data remaining elevated. The persistent elevated measurements of RSV WBS followed the weekly regional RSV case numbers (Supplementary Figure S2). In Hamilton, no significant divergence was observed between pediatric RSVH, the regional RSV cases and RSV WBS measurements.

## 4. Discussion

This study is among the first to compare real-time municipal RSV WBS data with clinical measurements of RSV disease prevalence and burden in the community. Monitoring RSV in municipal wastewaters over 31 weeks in 2022 and 2023 revealed a signal detection of RSV 12 days in advance of pediatric RSVH in two Canadian cities with similar clinical and wastewater testing regiments. In cities of Ottawa and Hamilton respectively, RSV WBS identified geographic variations in the rise of community RSV activity, at 36 days and 12 days, prior to the provincial RSV season start date and initiation of monthly government-funded palivizumab RSV immunoprophylaxis for at-risk infants and young children across the province of Ontario. Regional integration of WBS and clinical surveillance data revealed differences in RSV transmission patterns among the pediatric and adult populations between the two cities. In Ottawa, pediatric RSVH decreased while RSV WBS data remained elevated alongside regional RSV case numbers, suggesting a significant shift in RSV infections from the pediatric to the adult population and consistent with previous studies indicating relatively delayed adult hospitalization peaks (27).

RSV meets the three key criteria outlined by the CDC for pathogen targets in wastewater surveillance: public health significance, analytic feasibility of wastewater monitoring, and usefulness in informing infection prevention actions (28). Disproportionately impacting the very young and the older individuals, RSV is both the most common global causes of acute lower respiratory tract infection and the most common pathogen detected in infants and young children hospitalized with respiratory illness. Annual RSV seasonal outbreaks confer significant morbidity and mortality, frequently exceeding pediatric acute care service capacity (3). In addition, like influenza, there is considerable and under-recognized RSV-related morbidity and mortality in the older individuals (29).

There are several novel elements related to our study. First, two jurisdictions with similar approaches to the monitoring and management of seasonal pediatric RSV were prospectively compared. Second, comprehensive pediatric hospitalization and real-time wastewater surveillance data were assembled and concurrently fed back to an interdisciplinary group of environmental engineers and scientists along with clinical and public health experts. Third, clinical and environmental indicators were evaluated to determine optimal metrics for reliable and reproducible wastewater and clinical surveillance that may be generalizable to similar jurisdictions elsewhere. Finally, RSV WBS was performed to provide reliable, reproducible, and thus actionable results. In particular, our study demonstrates the analytical feasibility of near-real time RSV WBS that consistently precedes pediatric hospitalization data until the latter had peaked and can be used to more accurately determine the start of the RSV season. The advancements in RSV WBS were accomplished by applying a sampling frequency that was equal to or above a minimum recommendation of three samples per week (19), by implementing a 24-h turnaround time for sample processing and data analysis, and a by using solids-based analytical methods to measure RSV in wastewaters (resulting in low assay limits of detection and quantification) (22). Further, stringent RWV WBS quality assurance and quality control criteria were applied in the study to limit the effects of dilution, inhibition, and extreme weather events on WBS data.

Prior to the COVID-19 pandemic, RSV epidemics were highly predictable in frequency and intensity, with most cases in the Northern Hemisphere occurring between November and April. While relatively few RSV cases were reported during the 2020–2021 and 2021–2022 seasons, a resurgence was anticipated and duly realized in the autumn of 2022, with, as outlined above, wastewater indicators leading clinical indicators by nearly 2 weeks. This is in concordance with the lead time seen for WBS of influenza (30), and slightly longer than the 7 days lead time reported for RSV in the literature (18). This difference could be due to the fact that the clinical data documented by Koureas et al. used a broader case definition, influenza-like illness, which could include respiratory viruses with shorter incubation periods. Furthermore, RSV WBS is shown to capture the impact of geography on regional variations in the incidence of RSV, most pronounced between east–west and north–south (31), showing a 24-day difference in RSV onset between Ottawa (45° 24′ 40.21″ N, −75° 41′ 53.23″ W) and Hamilton (43° 15′ 0.40″ N, −79° 50′ 58.67″ W) (Supplementary Figure S1).

Most importantly, our findings suggest a potential role for real-time RSV WBS to meaningfully impact health policy decisions around timing of initiation of RSV immunoprophylaxis to reduce the burden of severe pediatric RSV disease and optimize regional healthcare resource allocation (32). In Canada, the RSV season definition and subsequent dosing of palivizumab among at-risk infants and children under 2 years of age is decided at the provincial level. Palivizumab is allocated to and administered in designated healthcare facilities based on the RSV season start and end dates. In Ontario, the RSV season definition is based on local RSV epidemiology, informed by the number of children hospitalized and regional test positivity (33). However, variations in the incidence of RSV across locales in the province do not always align with the provincial start date for the initiation of monthly RSV prophylaxis programs. RSV WBS provides an opportunity to transition from the current approach of a single provincial start date to multiple regional start dates, which would be at once novel and potentially cost-saving by enhancing timely detection and protection of at-risk children across the province and leading to fewer RSVHs and medically-attended emergency room or outpatient RSV infections. Given the surge of inter-seasonal RSV transmission since the COVID-19 pandemic, restricted access to clinical testing, and recognition that RSV immunoprophylaxis is most effective when initiated just before or near the start of the RSV season, real-time regional-level RSV WBS provides an economic, equitable, population-level early warning measurement of community RSV incidence to inform timely initiation of RSV immunoprophylaxis for at-risk infants and young children. Beyond its contributions to the prevention of severe disease, RSV WBS can be of considerable value as healthcare facilities plan for local surges in capacity demands and staffing requirements needed to care for children and adults with severe RSV infections.

More broadly, the WBS strategy can be used in communities worldwide to gage the prevalence of RSV in the population and the impact of novel monoclonal antibodies such as nirsevimab and emerging RSV vaccines. However, prior to adoption, the cost utility of the WBS maneuver needs to be firmly established. An updated Canadian model has been recently developed to evaluate the cost-effectiveness of palivizumab in 29–35-weeks’ gestational age infants who are at moderate to high risk of RSV-related hospitalization (34). The overall structure and decision tree of the current model can be adapted and validated for the economic evaluation of WBS to justify its use for the early detection of the RSV season with concurrent implementation of prophylaxis.

Several limitations of our study also merit consideration. First, we were limited in making inferences about the cause underlying the decoupling of pediatric RSVH and RSV WBS in late-November, as clinical RSV data do not capture the overall prevalence in the community. Presumably, this divergence reflected a shift in the burden to the adult population, given similar trajectories with regional laboratory data; however, provincial surveillance of adult RSVH was only initiated in late-November 2022 and not publicly reported. This highlights the need for more work to be done on understanding RSV transmission dynamics within populations as the season progresses. Moreover, if RSV surveillance was able to incorporate primary and secondary care surveillance systems, similar to the Global Influenza Surveillance and Response System (GISRS) (35), the lead time of RSV WBS would most likely be reduced. Third, our WBS system was already in place for SARS-CoV-2 and the existing platform was streamlined for RSV detection. In regions without an existing wastewater surveillance system, establishing sample collection and transportation logistics can pose challenges and significant initial planning and capital costs. Onboarding of remote communities presents even greater challenges due to constraints such as limited numbers of trained professionals, a notable barrier to implementation in these environments (35, 36). However, existing wastewater surveillance sites could serve as sentinel nodes for RSV WBS, with priority given to sites with geographic representativeness (e.g., urban and rural areas across the province). Additionally, only one season of RSV WBS data was available to test the definition of the start date by clinical and environmental criteria. Future RSV seasons will help consolidate and optimize the proposed criteria. Finally, further study is required to determine the validity of results in locations where minimum sampling frequency is not met.

This study demonstrates the potential for RSV WBS as a real-time surveillance tool to gage community-level RSV burden that more accurately and quickly identifies the start of the RSV season. The economic nature of WBS also provides an opportunity to provide a granular regional approach to RSV prophylaxis, to more effectively reduce the burden of illness among infants and children. WBS can also proactively facilitate local-level decisions around public health messaging on the prevention of respiratory illness, and control measures to reduce transmission in at-risk settings. This will help to streamline hospital resource allocation and promote timely initiation of seasonal RSV immunoprophylaxis for at-risk infants and young children. Finally, the unanticipated divergence of the WBS signal from traditional clinical indices will likely shed new light on the transmission dynamics of RSV in the community.

## Data availability statement

The datasets presented in this study can be found in online repositories. The names of the repository/repositories and accession number(s) can be found at: www.ottawapublichealth.ca/flureport. The data from the city of Hamilton can be found at this link https://www.hamilton.ca/people-programs/public-health/diseases-conditions/influenza-flu-respiratory-syncytial-virus-rsv.

## Author contributions

EM: Conceptualization, Formal analysis, Investigation, Methodology, Supervision, Validation, Visualization, Writing – original draft, Writing – review & editing. LP: Writing – review & editing. FG: Data curation, Writing – review & editing. SW: Conceptualization, Methodology, Writing – review & editing. NH: Formal analysis, Writing – review & editing. PD’A: Resources, Writing – review & editing. MK: Resources, Writing – review & editing. TN: Writing – review & editing. WE: Formal analysis, Writing – review & editing. BH: Writing – review & editing. ER: Writing – review & editing. CR: Data curation, Writing – review & editing. AM: Writing – review & editing. JW: Data curation, Writing – review & editing. CH: Data curation, Writing – review & editing. BP: Data curation, Writing – review & editing. RD: Conceptualization, Data curation, Formal analysis, Funding acquisition, Investigation, Methodology, Project administration, Resources, Supervision, Validation, Visualization, Writing – review & editing. NT: Conceptualization, Data curation, Formal analysis, Investigation, Methodology, Project administration, Supervision, Validation, Visualization, Writing – review & editing.
