# Corrigendum: Wastewater-based surveillance identifies start to the pediatric respiratory syncytial virus season in two cities in Ontario, Canada

**DOI:** 10.3389/fpubh.2024.1354693

**Published:** 2024-01-25

**Authors:** Elisabeth Mercier, Lakshmi Pisharody, Fiona Guy, Shen Wan, Nada Hegazy, Patrick M. D'Aoust, Md Pervez Kabir, Tram Bich Nguyen, Walaa Eid, Bart Harvey, Erin Rodenburg, Candy Rutherford, Alex E. Mackenzie, Jacqueline Willmore, Charles Hui, Bosco Paes, Robert Delatolla, Nisha Thampi

**Affiliations:** ^1^Department of Civil Engineering, University of Ottawa, Ottawa, ON, Canada; ^2^Hamilton Health Sciences, McMaster Children's Hospital, Hamilton, ON, Canada; ^3^Research Institute, Children's Hospital of Eastern Ontario, Ottawa, ON, Canada; ^4^Hamilton Public Health Services, Hamilton, ON, Canada; ^5^Hamilton Regional Virology Laboratory, Hamilton, ON, Canada; ^6^Department of Pediatrics, Children's Hospital of Eastern Ontario, Ottawa, ON, Canada; ^7^Department of Pediatrics, University of Ottawa, Ottawa, ON, Canada; ^8^Ottawa Public Health, Ottawa, ON, Canada; ^9^Department of Pediatrics (Neonatal Division), McMaster University, Hamilton, ON, Canada

**Keywords:** respiratory syncytial virus, wastewater-based surveillance, pediatric hospitalization, community incidence, season start date, active immunization, palivizumab, hospital-level preparedness

In the published article, there was an error in the **Conflict of interest statement**. The correct Conflict of interest statement appears below.

