# Supplementary material for: Wastewater-based surveillance identifies start to the pediatric respiratory syncytial virus season in two cities in Ontario, Canada
Source: Front Public Health. 2023 Sep 26;11:1261165. doi: 10.3389/fpubh.2023.1261165 (PMC10566629; doi:10.3389/fpubh.2023.1261165)
Supplement: Supplementary file 1 [file Data_Sheet_1.docx]

Supplementary Material

# WBS site description and sewershed demographic information

WBS was performed by collecting wastewater samples from the city of Ottawa’s Robert O. Pickard Environmental Centre, Ottawa’s sole water resource recovery facility (WRRF), and the Woodward WRRF, one of two WRRF’s in the city of Hamilton. Both WRRFs are connected to sewer network, a sewershed, that collects wastewater from approximately 91% (910,000/1,000,000 for Ottawa and 512,000/561,780 for Hamilton) of the population of each city. Additional information regarding the population serviced, flows and some demographic information is shown in Table S1.

|  | **City of Ottawa WRRF** | **Woodward WTTP, city of Hamilton** |
| --- | --- | --- |
| Population (approx.) | 910,000 | 512,000 |
| number of households (approx.) | 396,000 | 222,810 |
| Mean age of population | 40.0 | 41.5 |
| Average daily flow (m^3^/day) | 430,000 | 263,280 |

##

**Supplementary Table S1.** Approximate sewershed populations, number of households in the sewershed, mean age of the population living within the sewershed, and average dry daily flow of the city of Ottawa and Hamilton WRRFs.

# WBS PCR RT-qPCR Primers, probes and standard material used in this study

Primers, probes and standard material used in the study to quantify respiratory syncytial virus (RSV) and pepper mild mottle virus (PMMoV) are shown in Table S2.

| **Primer/probe (supplier)** | **Sequence** | **Reference** |
| --- | --- | --- |
| RSV forward primer (IDT) | CTCCAGAATAYAGGCATGAYTCTCC | (1) |
| RSV reverse primer (IDT) | GCYCTYCTAATYACWGCTGTAAGAC | (1) |
| RSV probe (IDT) | TAACCAAATTAGCAGCAGGAGATAGATCAG (5′HEX/ZEN/3′IBFQ) | (1) |
| PMMoV forward primer (ABI) | GAG TGG TTT GAC CTT AAC GTT GA | (2) |
| PMMoV reverse primer (ABI) | TTG TCG GTT GCA ATG CAA GT | (2) |
| PMMoV probe (ABI) | 6-FAM-CCT ACC GAA GCA AAT G-MGB | (2) |
| RSV G-block | CTCCAGAATACAGGCATGACTCTCCTGATTGTGGGATGATAATATTATGTATAGCAGCATTAGTAATAACCAAATTAGCAGCAGGAGATAGATCAGGTCTTACAGCTGTGATTAGGAGAGC | Designed in-house |

**Supplementary Table S2.** WBS RT-qPCR primers, probes and standard material utilized in this study.

# WBS RT-qPCR analysis and cycling conditions

All PCR reactions performed in this study were singleplex, TaqMan one-step RT-qPCR experiments. Samples were run in triplicate using a CFX96 touch real time thermocycler (Bio-Rad, Hercules, CA). PCR cycling conditions for all assays performed in this study are described below in detail in Table S3. Samples were quantified using five-point gradient dilutions with a g-block for RSV (Table S2).

The limit of detection of the RT-qPCR assay for RSV was determined by evaluating the least number of copies per reaction being detected with a detection rate of ≥95% (<5% false negatives), as recommended by the MIQE guidelines(3). Furthermore, samples were discounted if the following experimental conditions were not met: i) the standard curves had an R^2^ ≥ 0.95, ii) the copies/reaction were in linear dynamic range of the standard curve and iii) the primer efficiency lay between 90% to 120%. Furthermore, sample replicates with values greater than 0.5 standard deviations beyond the average of the triplicates were discounted.

|  | **RSV** | **PMMoV** |
| --- | --- | --- |
| **RT-qPCR conditions** | Reverse trans.: 5 min. @ 50°C, 1 cycle  Initial denat.: 20 sec. @ 95°C, 1 cycle  Denaturation: 3 sec. @ 95°C, 45 cycles  Anneal/ext.: 30 sec. @ 60°C, 45 cycles | Reverse trans. : 5 min. @ 50°C, 1 cycle  Initial denat.: 20 sec. @ 95°C, 1 cycle  Denaturation: 3 sec. @ 95°C, 45 cycles  Anneal/ext. : 30 sec. @ 60°C, 45 cycles |
| **Primer and probe concentrations** | 500 µM (primers)  200 µM (probes) | 500 µM (primers)  125 µM (probes) |
| **Supermix used and total reaction volume** | 1-Step Fast Virus (2.5 µL), 10 µL | 1-Step Fast Virus (2.5 µL), 10 µL |
| **Replicate exclusion threshold** | Ct ≥ 0.5 | Ct ≥ 0.5 |
| **Standard curve QA/QC** | R^2^ ≥ 0.95,  90% ≤ Efficiency ≤ 120% | R^2^ ≥ 0.95,  90% ≤ Efficiency ≤ 120% |
| **Other QA/QC performed** | Extraction blank, no extrapolation of values, negative controls | Extraction blank, no extrapolation of values, negative controls |

**Supplementary Table S3.** List of WBS RSV RT-qPCR thermal cycling conditions, primer and probe concentrations, and quality assurance and quality controls (QA/QC) controls employed during this study.

# Sanger sequencing of WBS RT-qPCR amplicons

The specificity of amplicons generated for various targets in this study was confirmed via Sanger sequencing of the DNA amplicons resulting from quantitative RT-qPCR analysis PCR was performed using Q5® High-Fidelity DNA Polymerase with 1 µl of RT-qPCR amplicons cleaned up using (QIAquick PCR kit – QIAgen 28104) as the starting template. The initial PCR was performed as follows: initial denaturation at 98°C (30 seconds), amplification [98C (10 sec) +55C (30 sec)+72C (30sec)] x 35 cycles and final extension 72C (2 min). The amplified products were then run on a 3% agarose gel at 100V to separate the amplicons. The amplicon band observed at the appropriate location the respiratory syncytial virus (RSV) N amplicon was located at 121 base pairs) was then cut and gel extracted using Monarch® DNA Gel Extraction Kit (New England Biolabs, MA, USA) as per the manufacturer’s instructions. The purified amplicon product was then sequenced by Sanger Sequencing at the Ottawa Hospital’s Research Institute (OHRI) StemCore Sequencing Facility using an ABI Prism 3730 DNA Sequencer (Applied Biosystems, MA, USA). The sequences were compiled and edited using BioEdit (ver.7.2) 6 and sequence alignment was done by Clustal Omega7.

# WBS to pediatric RSVH lead time and Spearman’s *ρ*

Spearman’s Rank correlation coefficients (*ρ*) were calculated between the RSVH and WBS dataset when offsetting WBS forward in time, by one-day increments, up to 25 days (Table S4).

| **Location** | **Lead time of WBS to RSVH** | **Spearman’s *ρ*** |
| --- | --- | --- |
| **Ottawa** | 0 day | 0.86 |
|  | 5 days | 0.87 |
|  | 12 days | 0.90 |
|  | 18 days | 0.88 |
|  | 25 days | 0.87 |
| **Hamilton** | 0 day | 0.82 |
|  | 5 days | 0.85 |
|  | 12 days | 0.90 |
|  | 18 days | 0.87 |
|  | 25 days | 0.82 |

**Supplementary Table S4**. Lead time of WBS to pediatric RSVH and associated Spearman’s ρ in Ottawa and Hamilton.

# Geographical location of Ottawa and Hamilton


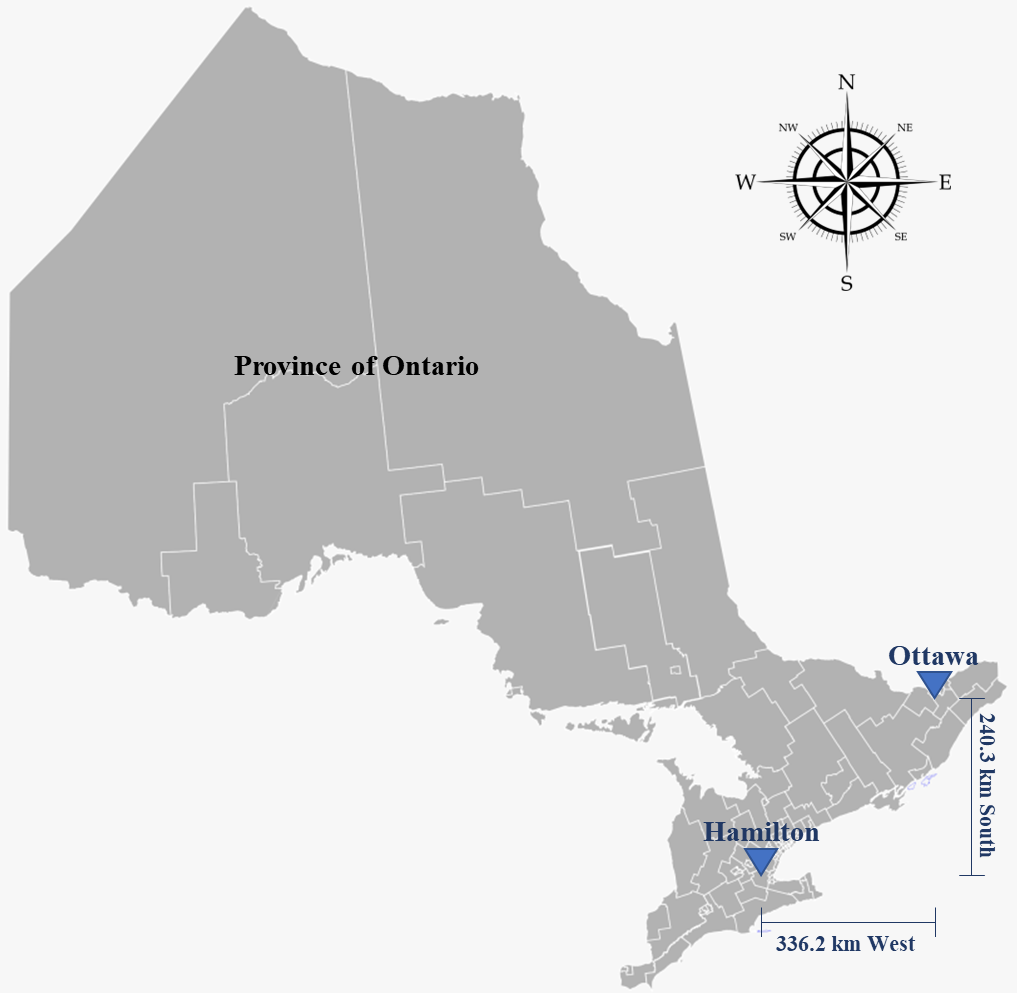


**Supplementary Figure S1.** Ottawa and Hamilton geographical location. The province of Ontario, Canada is shown in grey. The cities of Ottawa (45° 24′ 40.21″ N, -75° 41′ 53.23″ W) and Hamilton (43° 15′ 0.40″ N, -79° 50′ 58.67″ W), where RSV WBS was performed in this study, is identified by inverted triangles. The orientation depicts north at the top of the map.

# WBS and pediatric RSVH and regional RSV cases

**Supplementary Figure S2. 12-day shifted WBS measurements and pediatric RVSH and** **regional RSV cases of Ottawa and Hamilton.** 12 days shifted WBS RSV measurements (gene copies per grams of wastewater solids) for Ottawa and Hamilton in the upper and lower graphs, respectively. The wastewater measurements of both cities were collected in the sewersheds contained in the cities and described in Table S1. The population connected to and contributing to the sewershed of the two cities is contained within the surrounding regions. Weekly regional RSV cases represent the weekly number of laboratory-confirmed RSV cases including adult and pediatric inpatients and outpatients from the region and its surroundings. This encompasses the patients tested at any acute care sites within the regions of Ottawa and Hamilton, respectively including those requiring hospitalization (RSVH). For Ottawa, the regional RSV cases were sourced from the Eastern Ontario Regional Laboratory Association (EORLA) database, while the Hamilton Health Sciences adult and children’s hospitals provided the data for Hamilton. Weekly pediatric RSVH represents the weekly number of RSV hospitalization admissions, with laboratory-confirmed RSV test, to the Children’s Hospital of Eastern Ontario for the city of Ottawa and McMaster Children’s Hospital for the city of Hamilton.

# References

1. Hughes B, Duong D, White BJ, Wigginton KR, Chan EMG, Wolfe MK, Boehm AB. Respiratory Syncytial Virus (RSV) RNA in Wastewater Settled Solids Reflects RSV Clinical Positivity Rates. *Environ Sci Technol Lett* (2022) 9:173–178. doi: 10.1021/acs.estlett.1c00963

2. Haramoto E, Kitajima M, Kishida N, Konno Y, Katayama H, Asami M, Akiba M. Occurrence of pepper mild mottle virus in drinking water sources in Japan. *Appl Environ Microbiol* (2013) 79:7413–7418. doi: 10.1128/AEM.02354-13

3. Bustin SA, Benes V, Garson JA, Hellemans J, Huggett J, Kubista M, Mueller R, Nolan T, Pfaffl MW, Shipley GL, et al. The MIQE guidelines: Minimum information for publication of quantitative real-time PCR experiments. *Clin Chem* (2009) 55:611–622. doi: 10.1373/clinchem.2008.112797
